# Highly Reactive Isolevuglandins Promote Atrial Fibrillation Caused by Hypertension

**DOI:** 10.1016/j.jacbts.2020.04.004

**Published:** 2020-05-27

**Authors:** Joseph K. Prinsen, Prince J. Kannankeril, Tatiana N. Sidorova, Liudmila V. Yermalitskaya, Olivier Boutaud, Irene Zagol-Ikapitte, Joey V. Barnett, Matthew B. Murphy, Tuerdi Subati, Joshua M. Stark, Isis L. Christopher, Scott R. Jafarian-Kerman, Mohamed A. Saleh, Allison E. Norlander, Roxana Loperena, James B. Atkinson, Agnes B. Fogo, James M. Luther, Venkataraman Amarnath, Sean S. Davies, Annet Kirabo, Meena S. Madhur, David G. Harrison, Katherine T. Murray

**Affiliations:** a Department of Medicine, Vanderbilt University School of Medicine, Nashville, Tennessee; b Department of Pharmacology, Vanderbilt University School of Medicine, Nashville, Tennessee; c Department of Pediatrics, Vanderbilt University School of Medicine, Nashville, Tennessee; d Department of Pathology, Microbiology, and Immunology, Vanderbilt University School of Medicine, Nashville, Tennessee

**Keywords:** atrial fibrillation, atrial natriuretic peptide, B-type natriuretic peptide, hypertension, isolevuglandins, oxidative stress, preamyloid oligomers, 2-HOBA, 2-hydroxylbenzylamine, 4-HOBA, 4-hydroxylbenzylamine, AF, atrial fibrillation, ang II, angiotensin II, ANP, atrial natriuretic peptide, BNP, B-type natriuretic peptide, BP, blood pressure, ECG, electrocardiogram, G/R, green/red ratio, IsoLG, isolevuglandin, PAO, preamyloid oligomer, PBS, phosphate-buffered saline, ROS, reactive oxygen species

## Abstract

Oxidative damage is implicated in atrial fibrillation (AF), but antioxidants are ineffective therapeutically. The authors tested the hypothesis that highly reactive lipid dicarbonyl metabolites, or isolevuglandins (IsoLGs), are principal drivers of AF during hypertension. In a hypertensive murine model and stretched atriomyocytes, the dicarbonyl scavenger 2-hydroxybenzylamine (2-HOBA) prevented IsoLG adducts and preamyloid oligomers (PAOs), and AF susceptibility, whereas the ineffective analog 4-hydroxybenzylamine (4-HOBA) had minimal effect. Natriuretic peptides generated cytotoxic oligomers, a process accelerated by IsoLGs, contributing to atrial PAO formation. These findings support the concept of pre-emptively scavenging reactive downstream oxidative stress mediators as a potential therapeutic approach to prevent AF.

## Highlights

• IsoLGs are highly reactive lipid dicarbonyl metabolites that constitute a major component of oxidative stress-related injury, and they promote the formation of amyloid.
• In a hypertensive murine model, IsoLG adducts and PAOs developed in the atria, along with inducible AF.
• IsoLG and PAO accumulation and AF were prevented by the dicarbonyl scavenger 2-HOBA, but not by an inactive analog 4-hydroxybenzylamine.
• Mechanically stretched atrial cells generated cytosolic IsoLG adducts and PAOs that were prevented by 2-HOBA.
• Natriuretic peptides generated cytotoxic oligomers, a process accelerated by IsoLGs, contributing to atrial PAO formation.
• These findings identify a novel pathway during oxidative stress to increase AF susceptibility, and they support the concept of preemptively scavenging reactive downstream mediators as a potential therapeutic approach to prevent AF.

## Visual Abstract

**Figure:**
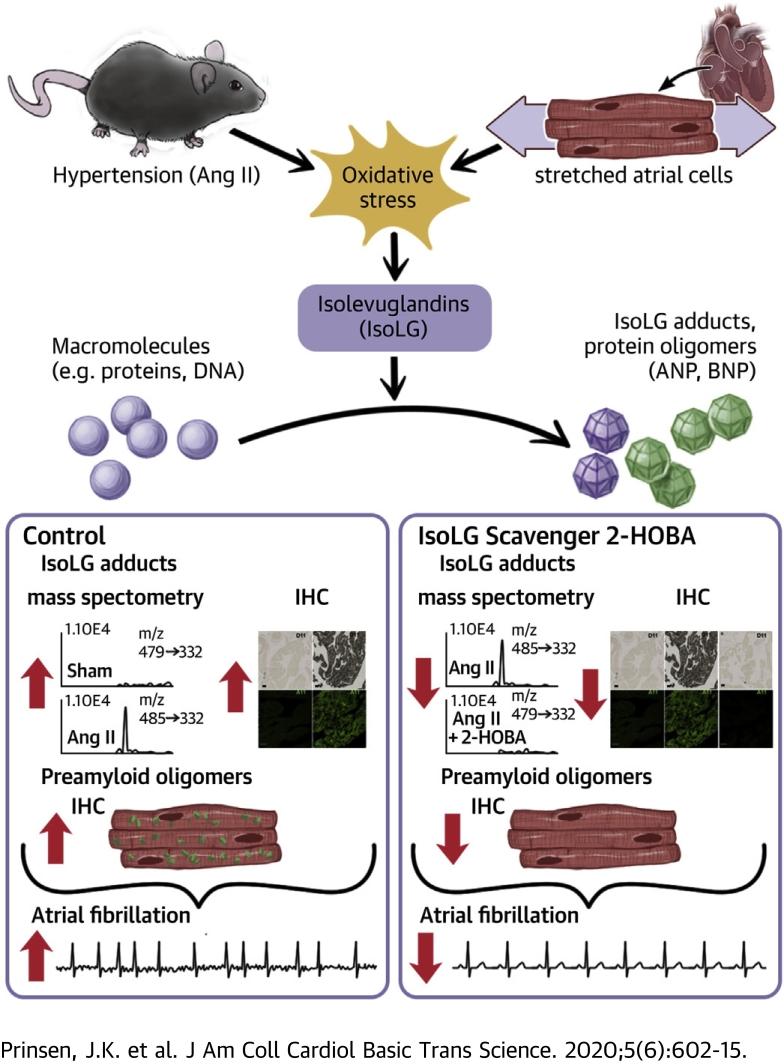

Atrial fibrillation (AF) is epidemic in the United States and worldwide, and it often results in devastating outcomes such as stroke and congestive heart failure (1). Nevertheless, currently available treatment designed to prevent or interrupt the AF substrate has met with only limited success, with the potential for serious adverse effects. Thus, there is a critical need for improved understanding of the underlying mechanisms causing AF and novel strategies to treat it.

There is abundant evidence linking oxidative stress and reactive oxygen species (ROS) directly to the pathogenesis and progression of AF (2). Inflammatory cells generate ROS, and inflammation-mediated AF is the most common and costly complication of cardiac surgery, as well as the mechanism of early recurrence following catheter ablation (3, 4, 5). In addition, multiple risk factors for AF, including hypertension, obesity, and aging, are mechanistically linked to oxidative stress (6,7). Unfortunately, “upstream therapy” targeting ROS levels directly with dietary antioxidants has been ineffective in clinical trials (8), in part because they fail to actually reduce oxidative injury in humans. Nonspecific ROS scavenging may also interfere with physiological ROS signaling.

Polyunsaturated fatty acid oxidation leads to the formation of highly reactive aldehydes. The most reactive of these products are dicarbonyl compounds known as isolevuglandins (IsoLGs) (also called γ-ketoaldehydes or isoketals [9,10]) (Figure 1). They adduct proteins almost instantaneously, causing misfolding and crosslinks (9). Tissue IsoLG adducts are elevated early in multiple diseases linked to inflammation and oxidative stress, including hypertension, obesity, atherosclerosis, and Alzheimer’s disease (11, 12, 13, 14, 15). Moreover, IsoLGs induce multiple effects that drive disease, including cytotoxicity, activation of inflammation and cytokine secretion, and acceleration of amyloidosis. In Alzheimer’s, misfolded protein amyloid β_1-42_ monomers coassemble initially to form soluble preamyloid oligomers (PAOs), now recognized to be the primary cytotoxic species correlating with disease progression rather than downstream amyloid fibril deposition (16,17). Importantly, IsoLGs markedly accelerate the oligomerization of amyloid β_1-42_ (18,19), providing a pathophysiological link between oxidative stress and proteotoxicity. As in the brain, amyloidosis develops in the human atrium with aging (20, 21, 22), and we recently identified PAOs in human atrial tissue (23).

**Figure 1:**
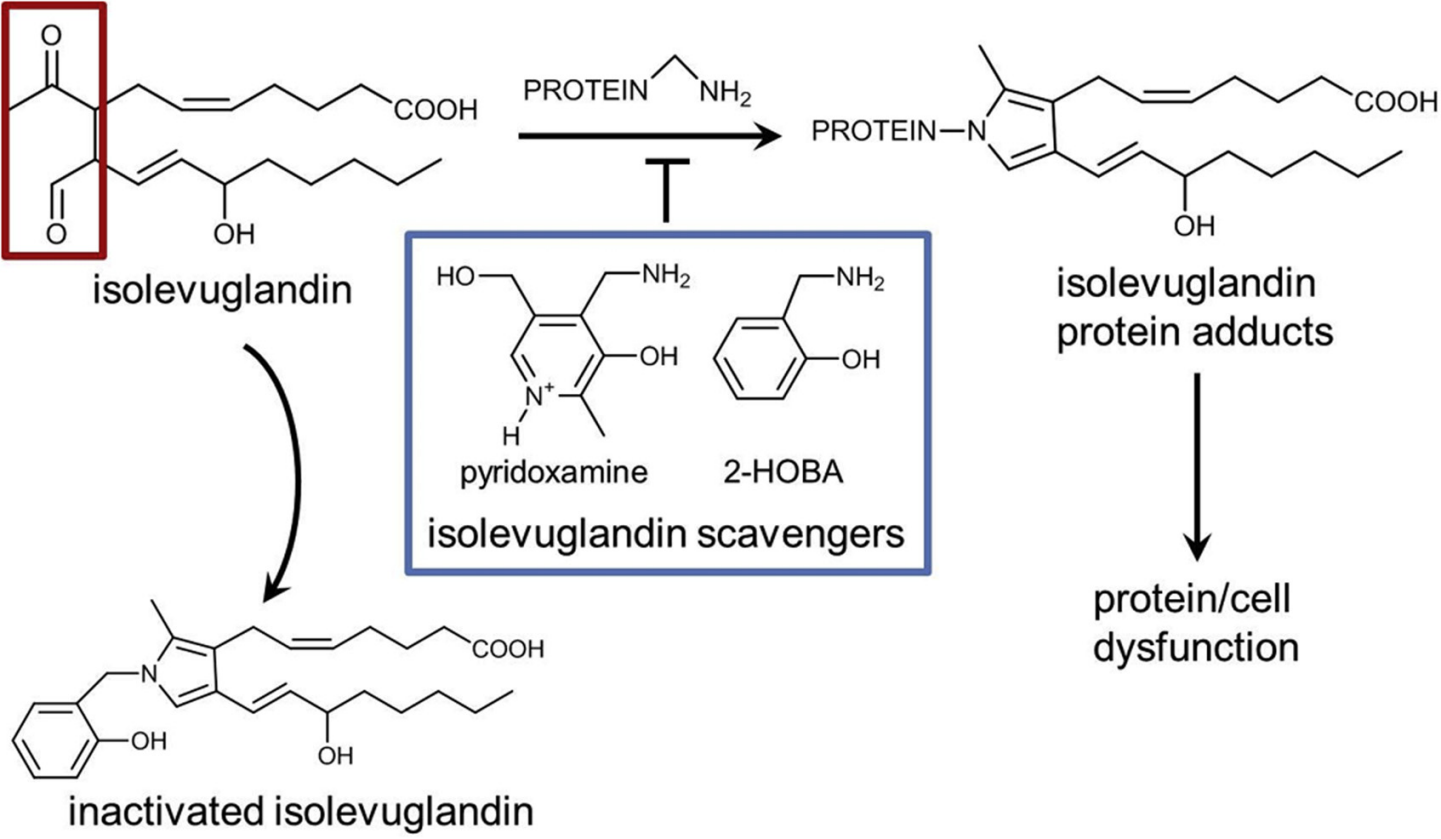
Mechanism of IsoLG Scavengers The 1,4-dicarbonyl **(red box)** IsoLGs interact rapidly with lysines to form lactam adducts and crosslinking of proteins. The phenolic amine pyridoxamine and its structural analog 2-HOBA **(blue box)** react with IsoLGs at a rate several orders of magnitude more rapidly than they react with lysines, thus serving as scavengers to prevent adduct formation. 2-HOBA = 2-hydroxylbenzylamine; IsoLG = isolevuglandin.

In a cellular model simulating AF, we previously found that rapid stimulation of atrial cells caused the formation of IsoLG adducts and protein oligomers within hours (24). We hypothesized that IsoLGs are molecular drivers of the AF substrate, constituting a novel mechanism to increase arrhythmia susceptibility. We chose a model of hypertension to test this hypothesis for several reasons. First, we found that the presence of protein oligomers in the human atrium was linked to hypertension (23). Second, considerable evidence implicates oxidative damage and inflammation in the development of hypertension (11,25). Third, it was recently demonstrated that IsoLG adducts are indeed formed during experimental hypertension, serving as neoantigens to promote dendritic and T-cell activation (11). In the present studies, we report that IsoLGs and PAOs develop in the atrium during murine hypertension and define a pathophysiological pathway linking oxidative stress and AF susceptibility. The findings identify downstream mediators of ROS-related injury as novel, alternative therapeutic targets for the prevention and treatment of AF.

## Methods

### Animal use

Male C57Bl/6J mice were obtained from Jackson Laboratory (Bar Harbor, Maine) and studied at 3 months of age. Hypertension was induced by continuous infusion of angiotensin II (ang II) (490 ng/kg/min) via osmotic minipumps (Alzet, Durect Corp., Cupertino, California) for 2 weeks. Blood pressure (BP) was monitored using tail cuff measurements preceded by acclimation. Oral 2-hydroxylbenzylamine (2-HOBA) (1 g/l), 4-hydroxylbenzylamine (4-HOBA) (1 g/l), or hydralazine + hydrochlorothiazide (320 mg/l and 60 mg/l, respectively) was delivered via drinking water (11).

### Atrial HL-1 cell culture

Atrial HL-1 cells were grown in Claycomb Medium (Sigma-Aldrich, Boston, Massachusetts) supplemented with 10% fetal bovine serum, 0.1 mmol/l norepinephrine, 2 mmol/l l-glutamine, and 0.1 mmol/l norepinephrine as described previously (24,26). Near-confluent/confluent cells (grown on a BioFlex Culture Plate for 48 h; Flexcell International, Burlington, North Carolina) were exposed to 10% cyclical stretch at a rate of 1 Hz for 24 h using the Flexcell FX-5000 Tension System (Flexcell International) (27).

### IsoLG adducts

Immunohistochemistry. Formalin fixed hearts were subjected to immunohistochemistry using an anti–IsoLG-lysyl adduct single-chain antibody (D11 ScFv) characterized previously (28). Images were captured using a high-throughput Leica SCN400 slide scanner automated digital image system from Leica Microsystems (Wetzlar, Germany). Whole slides were imaged at 20× magnification to a resolution of 0.5 μm/pixel. Tissue cores were mapped using Ariol Review software (Leica Biosystems Richmond, Richmond, Illinois). Because rapid stimulation of atrial cells can produce IsoLGs and PAOs, atrial tissue was analyzed for these parameters only from animals not subjected to electrophysiological studies.

### Quantitation by mass spectrometry

Flash-frozen atria were thawed in 4 ml of phosphate-buffered saline (PBS) containing indomethacin 100 μmol/l (Sigma-Aldrich) to prevent formation of IsoLGs via oxygenation by cyclooxygenase of arachidonic acid released during the process, and pyridoxamine 1 mmol/l (Sigma-Aldrich) as an IsoLG scavenger. Tissues were homogenized using a jaw homogenizer and tissue grind tubes, before centrifugation at 10,000 × g for 20 min at 4°C. The supernatant was collected for protein IsoLG adducts analysis.

Cells subjected to stretch, and control cells simultaneously cultured on BioFlex plates, but without stretch, were incubated with indomethacin and pyridoxamine, in 1 ml of PBS (pH 7.4) at 4°C for 30 min before harvest.

Protein concentrations in homogenized atria or cells were measured using a BCA Protein Assay kit (Pierce, Rockford, Illinois), and samples were subjected to complete enzymatic digestion to individual amino acids (15). A [^13^C_6_] internal standard was added, and the IsoLG-lysyl adducts were purified by solid-phase extraction and high-performance liquid chromatography before being quantified by liquid chromatography-tandem mass spectrometry assay using isotopic dilution as described previously (29).

### Quantitation of PAOs

Immunostaining was performed on optimal cutting temperature compound–embedded myocardial sections using a mouse monoclonal antibody specific for striated muscle (MF20, 1:10, Developmental Studies Hybridoma Bank, Iowa City, Iowa) to label myocardium, and a rabbit polyclonal antibody (A11, 1:3,000, EMD Millipore, Darmstadt, Germany) recognizing a conformational epitope common to all PAOs (30,31), with secondary goat anti*-*mouse Alexa 568–conjugated and donkey anti*-*rabbit Alexa 488–conjugated antibodies (Molecular Probes, Eugene, Oregon), respectively. Confocal images were acquired from the tissue sections, and a previously validated method was used to quantify the relative myocardial surface area (red) that contained PAOs (green), or green/red ratio (G/R), as a spatial representation of PAO burden in an atrial sample (32).

### Quantitation of fibrosis

Atrial samples were sectioned (5 μm) and stained using a standard Masson’s trichrome procedure to visualize collagen-rich tissue. Digitized images of the entire specimen were acquired using a high-throughput Leica SCN400 slide scanner imaged at 20× magnification (resolution 0.5 μm/pixel). Tissue cores were mapped using Ariol Review software, and the number of blue pixels was quantified as percentage of atrial myocardium.

### Alkaline congo red staining

Tissue sections were stained in Congo red solution using standard methods. Positive controls with known amyloid were stained and examined concurrently, and demonstrated apple green birefringence under polarized light. Experimental samples were evaluated by a pathologist (J.B.A., A.B.F.) blinded to experimental groups.

### Transesophageal electrophysiological studies

AF was induced during a transesophageal electrophysiological study by an operator blinded to treatment (33). Mice were anesthetized with isoflurane, and a surface electrocardiogram (ECG) (lead I) recording was obtained using subcutaneous 27-ga needles in each forelimb. The ECG channel was amplified (0.1 mV/cm) and filtered between 0.05 and 400 Hz. A 2-F octapolar electrode catheter (CIBer cath, NuMED, Hopkinton, New York) was positioned in the esophagus with placement adjusted until reliable atrial capture was obtained. Bipolar pacing was performed with a 1-ms pulse width at 3 mA. Baseline intervals were measured, and standard clinical electrophysiological pacing protocols were used to determine the atrioventricular effective refractory period and Wenckebach cycle length. AF inducibility was measured after burst atrial pacing (6 separate 15-s trains delivered at cycle lengths of 50, 40, 30, 25, 20, and 15 ms, respectively). AF was defined as development of rapid atrial activity with an irregularly irregular ventricular response lasting at least 1 s. The study was terminated for an animal if AF lasting 10 min occurred. Data were analyzed to quantitate total AF duration, representing the AF burden.

### Oligomer generation and western blot analysis

Synthetic α-atrial natriuretic peptide (ANP) (1-28) (SLRRSSCFGGRMDRIGAQSGLGCNSFRY-disulfide bond [C7-C23]) and B-type natriuretic peptide (BNP) (SPKMVQGSCFGRKMDRISSSSGLGCKVLRRH-disulfide bond [C10 to C26]) peptides were generated by RS Synthesis (Louisville, Kentucky). To test for oligomerization, peptide (10 μmol/l) was prepared in PBS buffer (pH 7.4) and incubated at room temperature for 24 h or up to 6 days. A separate sample was incubated for 24 h with either 2 to 4 molar equivalent of synthetic IsoLGs or dimethyl sulfoxide (vehicle) as described (24). After incubation, peptides were subjected to Western analysis. Briefly, equal amounts of peptide samples were resolved with a NuPage Bis-Tris 4-12% gel (Thermo Fisher Scientific, Waltham, Massachusetts) and transferred to a polyvinylidene difluoride membrane at 30 V for 1 h on ice. Blots were then blocked in 5% (w/v) nonfat milk in Tris-buffered saline 0.1% Tween 20 buffer and incubated in anti–α-ANP or anti-BNP antibody (1:500, Phoenix Pharmaceuticals, Burlingame, California) overnight. The antigens were detected by luminescence method (enhanced chemiluminescent kit Pierce ECL Substrate, Thermo Fisher Scientific), using horseradish peroxidase–conjugated secondary (goat anti-rabbit) antibody (1:5,000, Jackson ImmunoResearch, West Grove, Pennsylvania).

### Immunohistochemistry for natriuretic peptides

Adjacent frozen sections of atrium were immunostained for A11 and either ANP or BNP. For natriuretic peptides, immunostaining was performed using primary rabbit polyclonal anti–α-ANP (1-28; 1:200) and anti-BNP (1:500) antibodies (Phoenix Pharmaceuticals) as described previously for ANP (23).

### Cytotoxicity

BNP and ANP oligomers were generated by incubating the peptides at room temperature for 24 h, 3 days, and 7 days at a concentration of 30 μmol/l in PBS. Atrial HL-1 cells were plated at a density of 25,000 cells per 100 μl Claycomb Medium/well in a 96-well microplate (Perkin Elmer, Waltham, Massachusetts) pre-coated with gelatin and fibronectin, and incubated overnight (37°C, 5% CO_2_). Cells were then treated with BNP and ANP oligomers (0.45 μmol/l) for 24 h. At the end of the treatment, cytotoxicity of BNP and ANP oligomers on HL-1 cells were determined by measuring cellular ATP levels with an ATPlite assay (Perkin Elmer) according to the manufacturer’s instructions. Luminescence was measured using a Lumicount microplate reader (Global Medical Instrumentation, Ramsey, Minnesota).

### Statistical analysis

**Figure 2:**
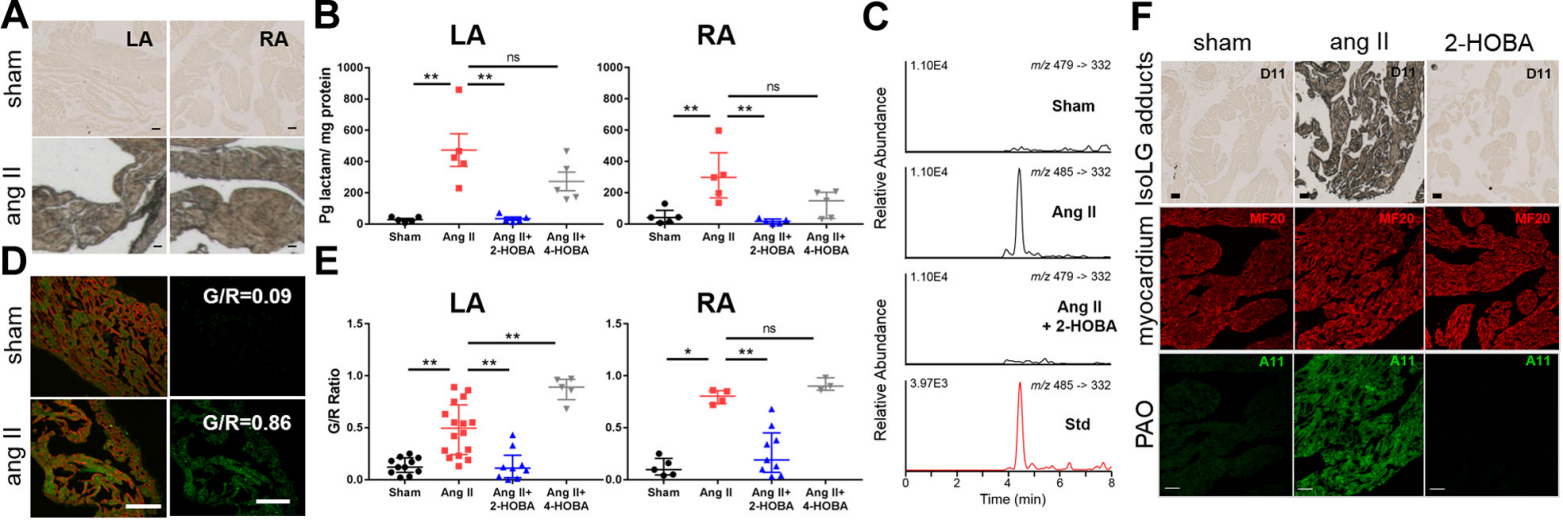
Hypertension Promotes the Formation of Atrial IsoLG Protein Adducts and PAOs, Which Is Inhibited by 2-HOBA **(A)** During ang II–mediated hypertension (ang II), striking accumulation of IsoLG protein adducts is demonstrated in left (LA) and right (RA) atria using immunolabeling with an anti–IsoLG-lysyl adduct antibody (D11 ScFv; n = 2, 4 for sham and ang II–treated mice, respectively; scale bars = 50 μm) compared with control mice (sham). **(B)** Summary data are shown for quantitation of IsoLG adducts in LA and RA using liquid chromatography-tandem mass spectrometry assay (mean ± SEM; n = 5 each; ∗∗p < 0.01 between indicated groups, ns is nonsignificant, nonparametric Mann-Whitney *U* test). **(C)** Representative mass spectrometry traces are shown for IsoLG adduct quantitation in LA from sham, ang II, and ang II+2-HOBA–treated mice, along with the internal standard in **red** (Std). **(D)** Confocal images are shown for myocardium **(red)** and PAOs **(green)** on the **left**, and PAOs localized to the myocardium on the **right**, from control and hypertensive mice, with PAO burden expressed as G/R values (scale bars = 20 μm). **(E)** Summary data are illustrated for oligomer burden in LA and RA (n = 11, 16, 9, 5 per group for LA; n = 5, 4, 9, 3 per group for RA; ∗p < 0.05, ∗∗p < 0.01 between indicated groups, nonparametric Mann-Whitney *U* test) (Scale bars = 50 μm). **(F)** 2-HOBA prevented development of IsoLG adducts **(upper panel)** and PAOs **(lower panel)** during ang II–mediated hypertension (also see **B** and **E**), whereas the inactive analog 4-HOBA had minimal effect. ang II = angiotensin II; PAO = preamyloid oligomer; other abbreviations as in Figure 1.

Data are expressed as mean ± SEM. For data with a skewed (non-normal) distribution, nonparametric Mann-Whitney *U* test was used to compare the differences in IsoLG adducts, G/R values, AF inducibility, and fibrosis (Figures 2B to 2E, 4A, and 4C, Supplemental Figures 1 and 2). The time and treatment effects on BP, as well as the modified effect of treatment by time, were analyzed using 2-way analysis of variance for repeated measures (Figure 4B). This is equivalent to a linear mixed-effects model with fixed effects on time, treatment, and their interaction and random intercept. The effect of incubation times on protein oligomer cytotoxicity was compared using 1-way analysis of variance with Tukey’s post hoc multiple pairwise comparison test (Figure 5C). A p value of <0.05 was considered statistically significant. Statistical analysis was performed using GraphPad Prism software version 7.02 (GraphPad Software, La Jolla, California).

### Study approval

All animal procedures were approved by the Vanderbilt Institutional Animal Care and Use Committee. Mice were housed and cared for in accordance with the Guide for the Care and Use of Laboratory Animals, U.S. Department of Health and Human Services.

## Results

### Hypertension causes formation of atrial IsoLGAdducts and PAOs, which is prevented by the Dicarbonyl scavenger 2-HOBA

Given that IsoLGs are formed in the vasculature during experimental hypertension (11), we hypothesized that this also occurs in the atrium. Immunohistochemistry was performed in the atria of mice rendered hypertensive by minipump infusion of angiotensin II (ang II) (27) using a single-chain antibody (D11 ScFv) that recognizes IsoLG-lysyl adducts on any protein (28). Hypertension caused diffuse IsoLG protein adduct accumulation in both the left and right atria (Figure 2A), which was absent in the atria of normotensive sham animals. This finding was confirmed by quantifying IsoLG adducts using mass spectrometry, with a significant increase in adduct formation in both atria of hypertensive animals (Figures 2B and 2C).

Small-molecule compounds, exemplified by 2-HOBA, have been identified that react with IsoLGs to pre-emptively scavenge these and closely related dicarbonyl mediators to prevent downstream protein modification (34,35). When mice were cotreated with 2-HOBA (starting 3 days before ang II infusion), the formation of IsoLG adducts during hypertension was prevented (Figures 2B and 2F). A separate group of hypertensive mice was treated with the related structural analog 4-HOBA, which is a very poor scavenger of IsoLGs (11,34). For these animals, IsoLG adduct levels were not significantly different from those seen in mice treated with ang II alone (Figure 2B), indicating the specificity of the effects of 2-HOBA to scavenge IsoLGs.

We have previously shown that amyloid-related protein oligomers develop in the atria of patients undergoing cardiac surgery, where the oligomers are linked to hypertension (23). To determine whether PAOs are formed in murine atrium during hypertension, we performed immunohistochemistry using a conformation-specific antibody (A11) recognizing PAOs derived from any protein irrespective of amino acid sequence (30). Compared with normotensive animals, hypertension led to significant accumulation of PAOs in both the left and right atria (Figures 2D and 2E). As for IsoLG adducts, this effect was abrogated by 2-HOBA (Figures 2E and 2F), whereas the inactive structural analog 4-HOBA failed to prevent PAO formation (Figure 2E).

### IsoLG adducts and PAOs develop early during hypertension

**Figure 3:**
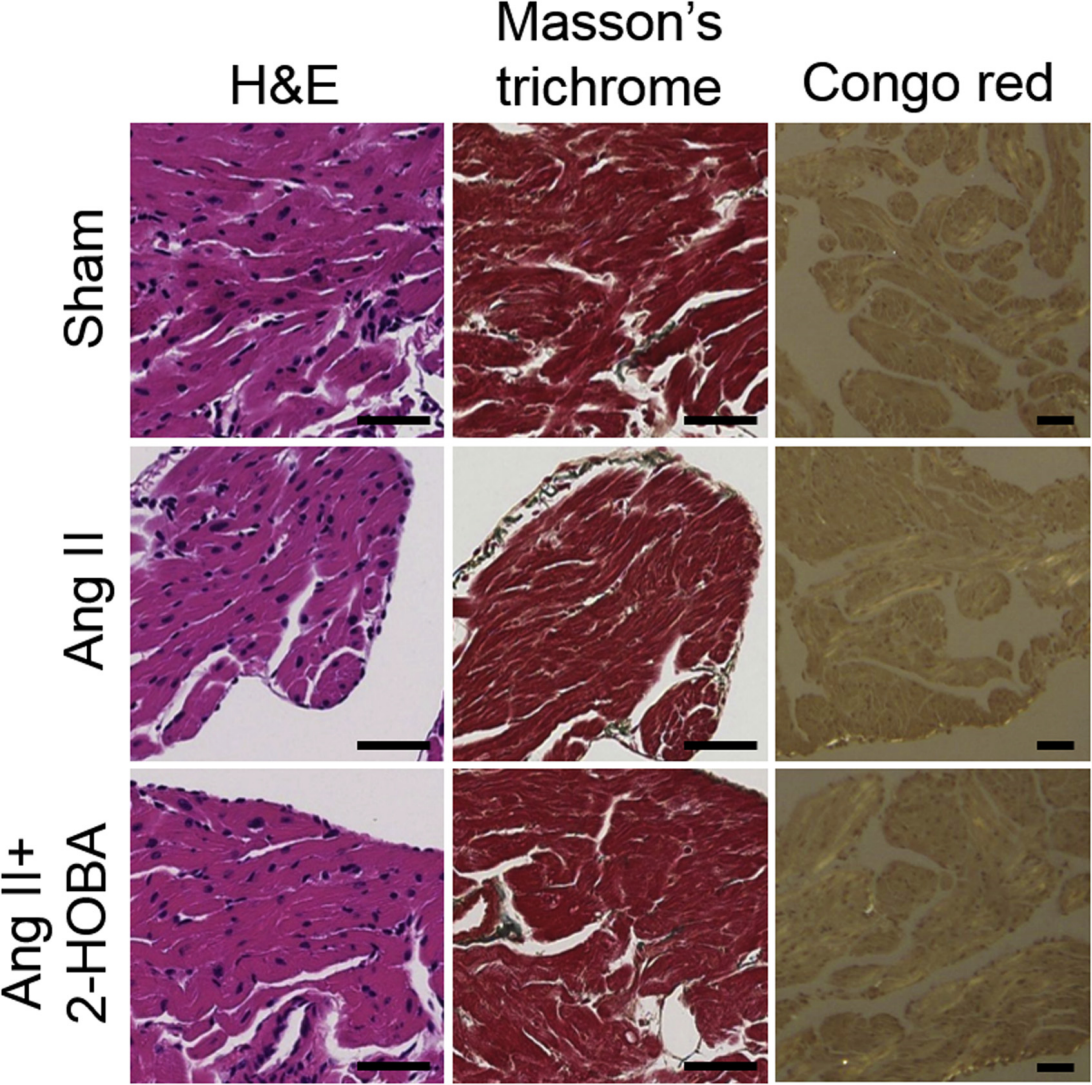
IsoLG Adducts and PAOs Develop at an Early Point During Hypertension, When Histological Abnormalities Are Absent For normotensive (sham), hypertensive (ang II), and 2-HOBA–treated hypertensive (ang II+2-HOBA) animals, **columns from left to right** display representative atrial images after exposure to hematoxylin and eosin (H&E), Masson’s trichrome, and Congo red stains. There was no evidence of myocardial structural abnormalities or amyloid, with minimal fibrosis that was similar between groups (see text). Scale bars = 50 μm. Abbreviations as in Figures 1 and 2.

Histochemical staining was performed to determine whether additional myocardial abnormalities were present in this model (Figure 3). Hematoxylin and eosin staining showed no difference in atrial histology between hypertensive and normotensive control mice. In addition, Masson’s trichrome staining demonstrated minimal fibrosis in sham, ang II–treated, and ang II+2-HOBA–treated animals (Supplemental Figure 2) (5.0 ± 0.6%, 5.0 ± 0.7%, and 4.6 ± 0.5%, respectively; n = 5 each), with no evidence of amyloid formation by Congo red staining. Thus, IsoLGs and PAOs occurred early in the pathogenesis of this hypertensive model before the development of significant atrial structural abnormalities.

### 2-HOBA suppresses hypertension-mediated atrial fibrillation

**Figure 4:**
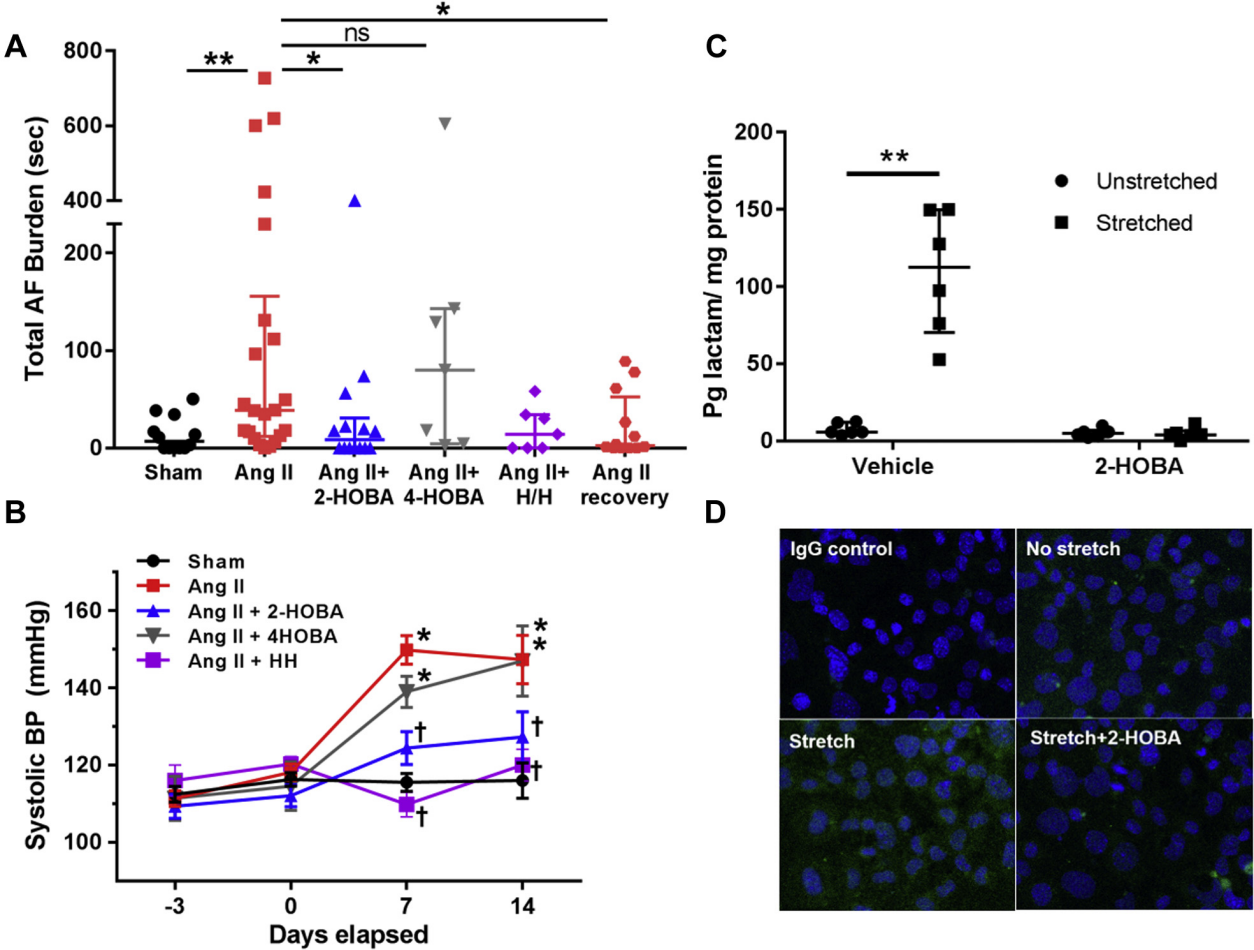
2-HOBA Prevented AF in Hypertensive Mice and Suppressed IsoLG Adduct and PAO Formation in Mechanically Stretched Atrial Cells **(A)** Total AF burden was increased in hypertensive (ang II) mice compared with controls (sham; n = 13, 22; ∗∗p < 0.01, nonparametric Mann-Whitney *U* test). During hypertension, cotreatment with 2-HOBA reduced AF burden, whereas the inactive structural analog 4-HOBA had no effect (ang II+2-HOBA, ang II+4-HOBA; n = 14, 7, respectively; ∗p < 0.05, nonparametric Mann-Whitney *U* test). Blood pressure normalization with hydralazine/hydrochlorothiazide (H/H) and cessation of ang II also led to a reduction AF (ang II+H/H, and ang II recovery; n = 7, 12, respectively; ∗p < 0.05, nonparametric Mann-Whitney test). **(B)** Summary data for systolic blood pressure are illustrated for the groups studied (∗p < 0.01 compared with sham, †p < 0.01 compared with ang II, 2-way analysis of variance for repeated measures). **(C)** Atrial HL-1 cells were subjected to either no stretch or stretch (10% at 1 Hz) for 48 h and analyzed by liquid chromatography-tandem mass spectrometry assay. Stretch caused robust development of IsoLG adducts, which was abrogated by 2-HOBA (n = 6 each; ∗∗p < 0.01, nonparametric Mann-Whitney *U* test). **(D)** Immunostaining demonstrates that atrial cells developed PAOs in response to stretch **(lower left)** compared with no stretch **(upper right)** or during stretch in the presence of 2-HOBA **(lower right)**. AF = atrial fibrillation; other abbreviations as in Figures 1 and 2.

AF susceptibility was investigated in control and hypertensive mice using transesophageal electrophysiological studies that employed rapid atrial burst pacing (33). Compared with control mice, the total amount or burden of inducible AF was significantly increased in hypertensive mice (Figure 4A). The AF substrate was reversible, with a 95% reduction in total AF burden within 2 weeks after stopping ang II (Figure 4A) (associated with a 70% reduction in BP (Supplemental Figure 1) (n = 12), providing further support that IsoLGs were generated early in the development of the AF substrate. Cotreatment with 2-HOBA significantly reduced AF burden compared with ang II alone (Figure 4A), whereas for mice receiving 4-HOBA, AF burden was comparable to that seen with animals receiving ang II alone. There were no effects of 2-HOBA on any ECG or electrophysiological parameters (Table 1). Taken together with the results shown in Figure 2, these findings demonstrate that ang II–mediated hypertension promotes the formation of atrial IsoLGs, PAOs, and AF susceptibility, with IsoLGs playing a critical role in the pathophysiological process.

**Table 1:** Intergroup Comparison of Electrophysiological Parameters

	Sham (n = 9)	Ang II (n = 14)	p Value	Ang II+2-HOBA (n = 10)	p Value∗
SCL, ms	127 ± 5	119 ± 3	0.25	117 ± 4	0.76
PR, ms	39 ± 1	38 ± 1	0.58	38 ± 1	0.55
QRS, ms	13 ± 1	13 ± 1	0.45	14 ± 1	0.89
QT, ms	43 ± 2	44 ± 1	0.99	41 ± 1	0.39
AVERP, ms	56 ± 2	54 ± 2	0.31	57 ± 1	0.14
WCL, ms	77 ± 2	76 ± 2	0.23	77 ± 1	0.16

Values are mean ± SEM.

2-HOBA = 2-hydroxylbenzylamine; AVERP = atrioventricular effective refractory period; SCL = sinus cycle length; WCL = Wenckebach cycle length.

∗ Comparison of angiotensin II (ang II) + 2-hydroxylbenzylamine (2-HOBA) with ang II.

### Atrial stretch causes IsoLG and PAO formation that is suppressed by 2-HOBA

In a separate cohort of mice receiving ang II, BP was normalized by the concomitant administration of hydralazine and hydrochlorothiazide, and this was associated with a low AF burden similar to that of sham-treated control mice (Figures 4A and 4B). To investigate the role of atrial myocyte stretch in the pathophysiological process, atrial HL-1 cells were cultured in the absence and presence of 10% cyclical stretch. Exposure to stretch caused a substantial increase in IsoLG adducts (Figure 4C), as well as the generation of protein oligomers (Figure 4D), and both effects were prevented in the presence of 2-HOBA. These findings point to a causative role for atrial cell stretch in the pathophysiology of AF susceptibility during hypertension.

### Isolevuglandins accelerate formation of cytotoxic natriuretic peptide oligomers, which contribute to hypertension-mediated PAOs in the atria

**Figure 5:**
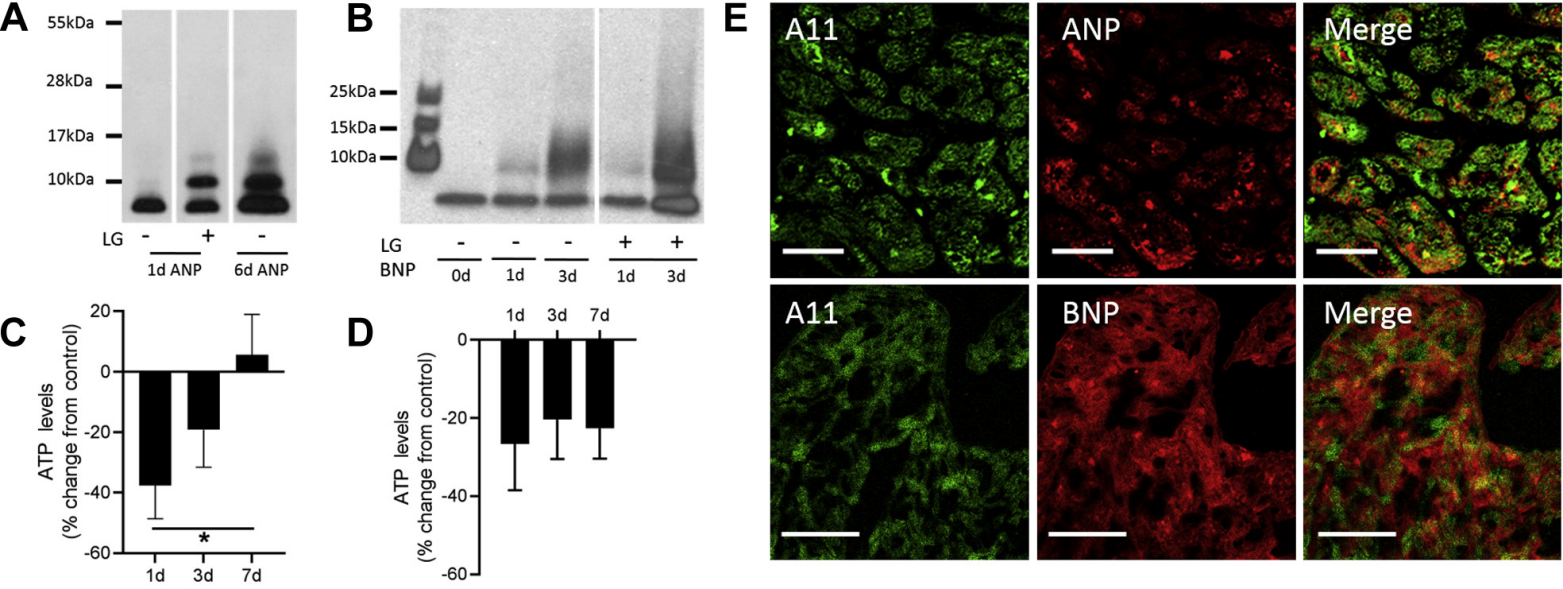
ANP and BNP Form Cytotoxic Protein Oligomers in Hypertensive Atria **(A)** Western blotting is shown following incubation of ANP peptide (10 μmol/l) at 22^o^C for 24 h or 6 days, compared with incubation with IsoLGs (synthetic 15-E_2_-IsoLG, 1 μmol/l) for 24 h, demonstrating time-dependent oligomerization that is markedly accelerated by IsoLGs. **(B)** Similar results are shown for BNP (10 μmol/l) following 0 to 3 days of incubation in the absence and presence of IsoLGs. **(C and D)** ANP and BNP (30 μmol/l) were allowed to oligomerize for 1, 3, and 7 days. Oligomers were incubated with atrial HL-1 cells (0.45 μmol/l for 24 h), followed by quantitation of cellular ATP production expressed as % change from control untreated cells. Upon exposure to oligomers, there was a reduction in ATP production indicative of cytotoxicity that declined significantly with increased oligomerization time for ANP (mean ± SEM; n = 5 independent experiments; ∗p < 0.05, 1-way analysis of variance with Tukey’s multiple comparison test). **(E)** Immunofluorescent labeling with A11 **(left)** and ANP- or BNP-specific antibodies **(middle)** was performed in adjacent 5-μm atrial sections from a hypertensive mouse (scale bars = 50 μm). Evidence of partial colocalization of natriuretic peptides with PAOs **(right)** is indicated by **lighter greenish yellow color**. ANP = atrial natriuretic peptide; ATP = adenosine triphosphate; BNP = B-type natriuretic peptide; other abbreviations as in Figures 1 and 2.

The amyloid-forming protein ANP is a prominent component in aging-related (senile) atrial amyloidosis, and some studies support the presence of BNP in these deposits as well (20,22,36). Given that ANP is a component of the PAOs that form in both human atrium and rapidly stimulated atrial cells (23,24), we investigated the role of natriuretic peptides in hypertension-mediated PAOs using several approaches. Purified ANP and BNP incubated at room temperature demonstrated time-dependent oligomerization, indicated by the development of additional higher molecular weight bands on Western blot analysis (Figures 5A and 5B). However, when incubated in the presence of IsoLGs, PAO formation was markedly accelerated. We then examined whether natriuretic peptide oligomers were detrimental to atrial cells. Both ANP and BNP oligomers reduced ATP production in atrial HL-1 cells, indicating cytotoxicity (Figures 5C and 5D). This effect was most pronounced for oligomers formed during a 1-day incubation, whereas cytotoxicity progressively declined with longer incubation times, most prominently for ANP (Figure 5C). This time course is analogous to that observed for amyloid β_1-42_ neuronal injury: as monomers coalesce to oligomers and subsequently to less toxic fibrils, PAO formation and associated cytotoxicity develops and then declines in a time- and concentration-dependent manner (17). Finally, adjacent sections of hypertensive mouse atria were immunostained for PAOs and either ANP or BNP, with results demonstrating evidence of partial colocalization of natriuretic peptides with atrial oligomers (Figure 5E). Taken together, these findings support a role for cytotoxic ANP and BNP oligomers as potential mediators of atrial pathophysiology during hypertension.

## Discussion

**Figure 6:**
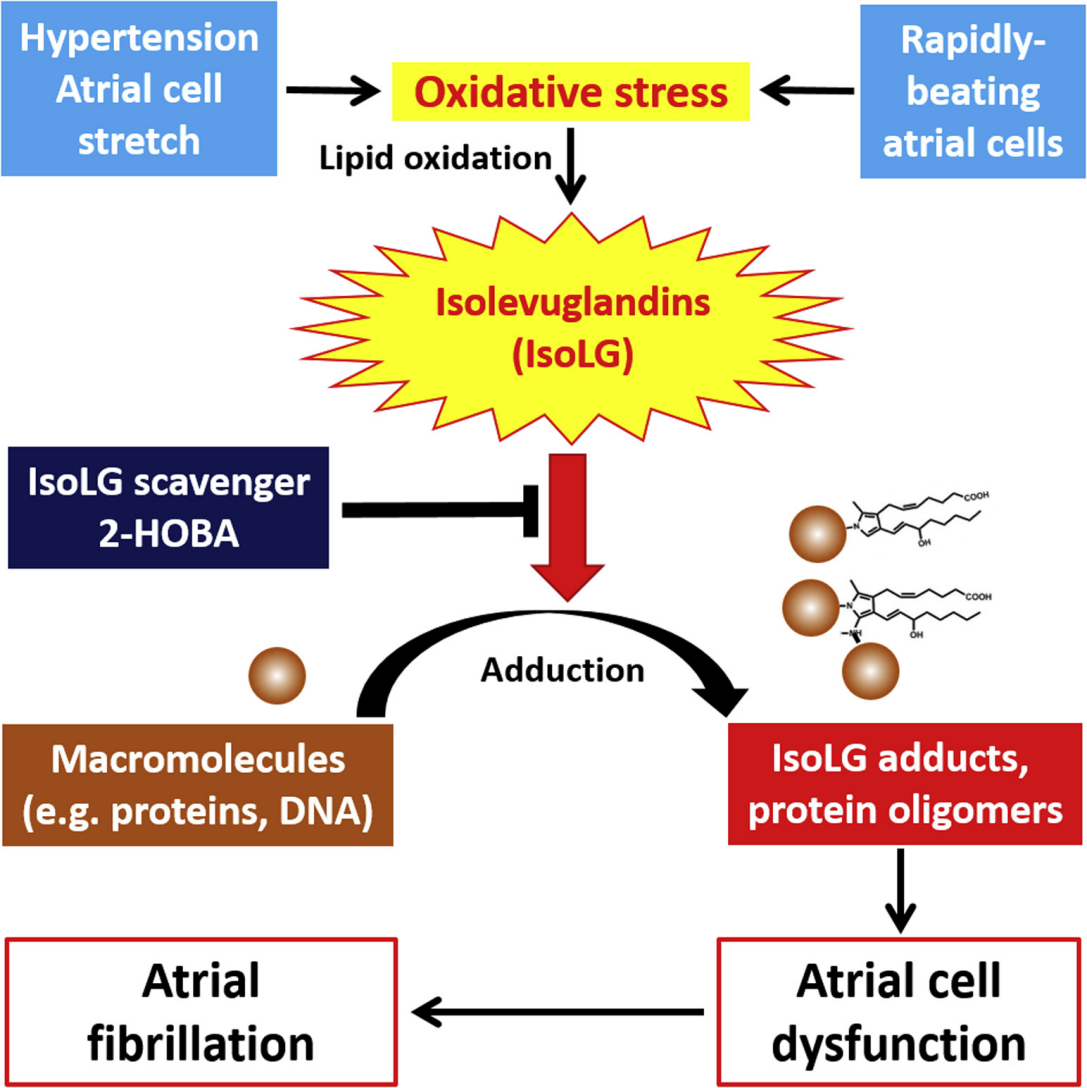
In Response to Hypertension, Oxidative Stress-Mediated IsoLGs Promote AF Susceptibility Hypertension and atrial cell stretch, as well as the rapid activation of atrial cells, causes oxidative stress and formation of highly reactive IsoLGs, that rapidly adduct and crosslink cellular proteins and other macromolecules. The generation of dysfunctional adducted proteins and protein oligomers promote atrial myocyte dysfunction to increase AF susceptibility. Abbreviations as in Figures 1 and 4.

As the most common sustained cardiac arrhythmia, AF constitutes a significant public health problem for which optimal medical therapies are lacking. Elucidating early mechanisms that increase AF susceptibility are critical to develop effective preventative and therapeutic strategies. In this study, we identified a novel role for highly reactive IsoLGs in the pathophysiology of hypertension-mediated AF. Using a murine model of hypertension, we found that atrial IsoLG adducts and cytotoxic protein oligomers were generated before histological abnormalities, associated with AF susceptibility that was reversible when BP declined. These detrimental effects were prevented by the dicarbonyl scavenger 2-HOBA, but not the ineffective analog 4-HOBA, confirming the specificity of this biochemical mechanism. Experiments in vitro and in vivo revealed a critical role of atrial myocyte stretch in the generation of IsoLG adducts during the pathophysiological process. These findings support the concept of pre-emptively scavenging reactive downstream mediators of oxidative stress, rather than targeting ROS generation per se, as a novel therapeutic approach to prevent AF (Figure 6).

With inflammation and oxidative stress, peroxidation of fatty acids generates multiple reactive aldehydes, including malondialdehyde (MDA), 4-oxo-2-nonenal, and IsoLGs (9,10,37,38). The toxicity of such compounds is markedly augmented by the presence of 2 carbonyl groups (C=O), and IsoLGs have a 1,4-dicarbonyl ring configuration that renders them extremely reactive (Figure 1) (9,34). These compounds react nearly instantaneously with proteins and are the most reactive products of lipid peroxidation identified to date (9). Indeed, they modify proteins so rapidly that they can only be detected in vivo as adducts rather than their unreacted form, in contrast to other lipid oxidation products.

IsoLGs form covalent adducts with amines, notably the epsilon amine of lysines in proteins, causing irreversible protein modifications. An intermediate in this reaction is also highly reactive, generating intramolecular crosslinks that cause dysfunction of proteins, including structures relevant to cardiomyocyte homeostasis, such as ion channels (39,40), HDL (13,14,41), mitochondria (42), histones (43), and proteasomes (44). IsoLGs can also adduct to DNA and phosphatidylethanolamines (45,46). Tissue IsoLG adducts are elevated early in animal models of cardiovascular risk factors, including hypertension, obesity, and hyperlipidemia (41), as well as atherosclerosis (13,14). They are also increased in other diseases linked to oxidative injury/inflammation, such as chronic ethanol exposure (47), pulmonary fibrosis (48), Alzheimer’s disease (15), and cancer (49). To date, IsoLG adducts identified in experimental models have emerged as critical mediators of oxidative injury in the brain during Alzheimer’s disease, and in the vasculature during hypertension and atherosclerosis (11,13,14,50).

Multiple risk factors for developing AF are associated with increased atrial pressure that promotes atrial tension/enlargement, and our results support a critical role for atrial cell stretch in the pathophysiological process. Atrial myocyte stretch triggers a generalized stress response, with activation of immediate early genes, dedifferentiation, activation of hypertrophic signaling cascades, and increased release/production of natriuretic peptides (51,52). Importantly, stretch of ventricular myocytes causes rapid production of superoxide (53). Similarly, in the present study, we found that atrial myocyte stretch causes IsoLG adduct formation, indicative of atrial ROS production. Prevention of AF susceptibility using a dicarbonyl scavenger is consistent with the concept that stretch-mediated oxidative stress is an early event in generating the AF substrate.

Diseases related to oxidative stress are increasingly linked to proteotoxicity as a contributing mechanism (16,31,54), in particular for neurological and cardiac dysfunction (16,17,31). The generation of atrial PAOs in this model is not unexpected on the basis of several considerations. First, the development of natriuretic peptide-related amyloidosis is almost universal in the aging human atrium (20, 21, 22). Second, we showed that IsoLGs markedly accelerate the oligomerization of ANP and BNP in vitro and in cells, yielding cytotoxic oligomers, as occurs with amyloid β_1-42_ (18,19). Finally, elevated concentrations of amyloidogenic proteins are a major factor that drives oligomer formation (55), and both local and systemic concentrations of natriuretic peptides are increased with stretch and rapid atrial contraction. Given that oxidative stress-mediated IsoLG formation promotes proteotoxicity in both the heart and brain, this provides a potential mechanism for the pathophysiological link between AF and dementia (56).

By targeting downstream mediators of ROS-related injury, dicarbonyl scavengers represent a totally novel therapeutic approach for diseases linked to oxidative stress. Contemporary antioxidants have been largely ineffective in such diseases, including AF. However, therapeutically used doses of antioxidants such as vitamin E and fish oil are not effective to reduce in vivo measures of oxidative injury (e.g., F_2_-isoprostanes, widely used sensitive markers of oxidative stress) (57, 58, 59). Dicarbonyl scavengers represent an alternative strategy to leave ROS generation intact, but to rapidly scavenge reactive lipid mediators as they form, rendering them inactive, so that they cannot interact with their biological targets. For 2-HOBA, structure–activity relationship assays demonstrated that the close proximity of the methylamine to the hydroxyl group (Figure 1) is key to scavenger potency (34,35). For the related analog 4-HOBA, this structural proximity is lost—hence, this compound is a very poor scavenger of dicarbonyls, enabling it to serve as a negative control. Importantly, 2-HOBA and its analogs are not antioxidants in that they do not react with O₂˙ˉ, OONO^−^, or H_2_O_2_ (11), and the reduction in IsoLG adduct levels has been attributed directly to the dicarbonyl scavenging effect, and not to inhibition of ROS production and/or lipid peroxidation. Although 2-HOBA reacts with IsoLG (a 1,4-dicarbonyl) much more rapidly than with MDA (a 1,3-dicarbonyl) or methylglyoxal (a 1,2-dicarbonyl), 2-HOBA is capable of scavenging these other dicarbonyls in vivo (11,60,61). 2-HOBA does not inhibit COX1 or COX2, and thus the production of physiological prostaglandins is preserved (62). To date, in vivo studies have demonstrated a beneficial effect of 2-HOBA in animal models of Alzheimer’s disease (50), hypertension (11), and atherosclerosis, with improvement in high-density lipoprotein function (13,14,41). Interestingly, 2-HOBA has also been shown to prolong the life span of *Caenorhabditis elegans* by ∼56% (63).

### Study limitations

A limitation of the study is that experiments were performed in a single murine model. The specifications of these mice were selected based on a previously published study that demonstrated accumulation of IsoLG adducts in the heart and aorta in this model (11), as proof of concept. In addition, male mice were chosen because the BP response to ang II in female mice is considerably reduced compared with males. Preliminary data in a mouse model of obesity demonstrated a similar beneficial effect of 2-HOBA to reduce AF susceptibility (12), supporting the potential generalizability of our findings to other conditions. Although cytotoxic protein oligomers are generated during murine hypertension, our findings do not prove a causative role for PAOs in the pathogenesis of hypertension-mediated AF. In addition, our results suggest that the atrial oligomers formed in this model are composed of additional protein components besides ANP and BNP, given that immunostaining for PAOs and natriuretic peptides demonstrates partial overlap. Clarifying the specific nature of injurious mediators and identification of other PAO-forming proteins is an important goal of future studies. Finally, although our data demonstrated a reduction in cytotoxicity with longer peptide incubation times for ANP supporting PAOs as the cytotoxic moiety, this was not observed for BNP. Nonetheless, these experiments were performed solely to assess oligomer cytotoxicity, rather than the kinetics of PAO/amyloid formation for the natriuretic peptides.

### Clinical implications

Our results provide evidence for a novel pathophysiological pathway in the genesis of the AF substrate. As highly reactive mediators of oxidative stress-related injury, IsoLGs are logical candidates for targeted inhibition using small molecule scavengers. By scavenging IsoLGs preemptively, dicarbonyl scavengers may represent a paradigm shift in pharmacological strategy to prevent injurious oxidative protein modification that can cause AF. Of note, 2-HOBA has been well tolerated in Phase 1 trials, with a Phase 2 trial to commence in the near future.

## Conclusions

Our findings demonstrate that hypertension promotes concomitant IsoLG and PAO accumulation along with arrhythmia susceptibility in the atrium, and they identify IsoLGs as a critical molecular component of this pathophysiological process. These findings provide a mechanistic link between hypertension, oxidative stress, proteotoxicity, and AF susceptibility.

Perspectives **COMPETENCY IN MEDICAL KNOWLEDGE:** The mechanism whereby oxidative stress increases AF susceptibility is not known. This paper identifies a novel molecular pathway by which highly reactive lipid dicarbonyl metabolites constituting a major component of oxidative stress-related injury are mechanistically linked to AF susceptibility during hypertension, a disease also linked to oxidative stress. Our findings also define a novel potential mechanism whereby oxidative stress promotes amyloid formation in the atria.

**TRANSLATIONAL OUTLOOK:** These findings identify a novel pathway during oxidative stress to increase AF susceptibility, and they support the concept of pre-emptively scavenging reactive downstream mediators, rather than targeting generation of reactive oxidative species per se, as a potential therapeutic approach to prevent AF. The scavenger 2-HOBA has been well-tolerated in initial Phase 1 clinical trials, and a Phase 2 trial will start within the next few months to examine its efficacy to prevent AF.
