# Supplementary material for: Highly Reactive Isolevuglandins Promote Atrial Fibrillation Caused by Hypertension
Source: JACC Basic Transl Sci. 2020 May 27;5(6):602–15. doi: 10.1016/j.jacbts.2020.04.004 (PMC7315188; doi:10.1016/j.jacbts.2020.04.004)

SUPPLEMENTAL MATERIAL

FIGURES

Figure 1: Blood pressure and AF burden in mice with discontinuation of angiotensin II. A. Systolic blood pressure is shown at baseline before starting of ang II infusion, after 2 weeks of ang II, and 2 weeks following cessation of ang II (n=12; $^{**}P<0.01$, one-way ANOVA with Tukey's multiple comparison test). **B.** AF burden is illustrated for the same mice after 2 weeks of ang II, and 2 weeks following cessation of ang II ($^{*}P<0.05$, non-parametric Mann-Whitney test).

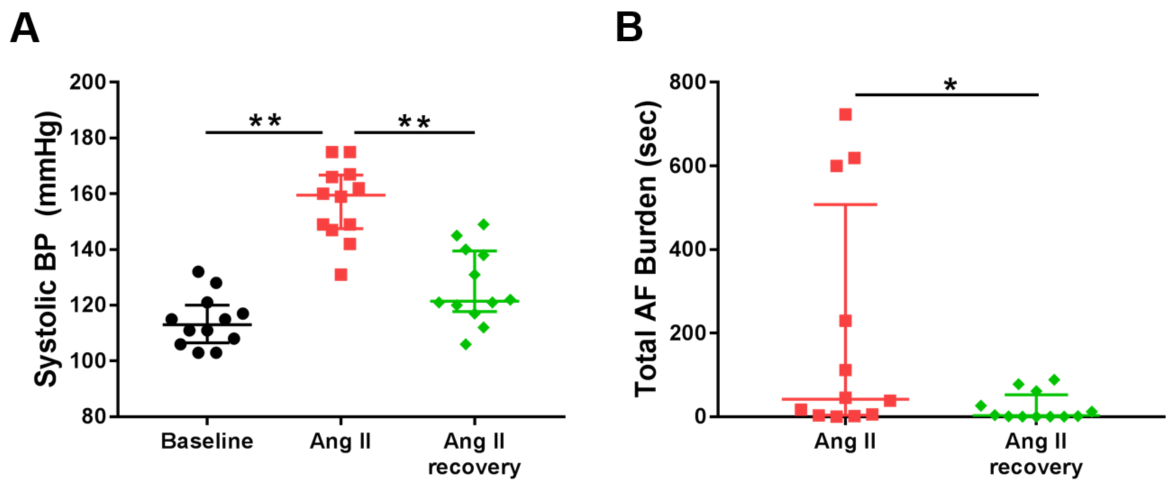

Figure 2: Quantitation of atrial fibrosis in mice treated with vehicle, angiotensin II, and angiotensin II+2-HOBA. A. Fibrosis expressed as % of myocardial area is shown for mice treated with vehicle (sham), ang II, and ang II+2-HOBA (n=5 mice in each group; data from left and right atria [LA, RA] are pooled). **B.** Data are presented with LA and RA shown separately ($^{*}P<0.05$, $^{**}P<0.01$, non-parametric Mann-Whitney test).

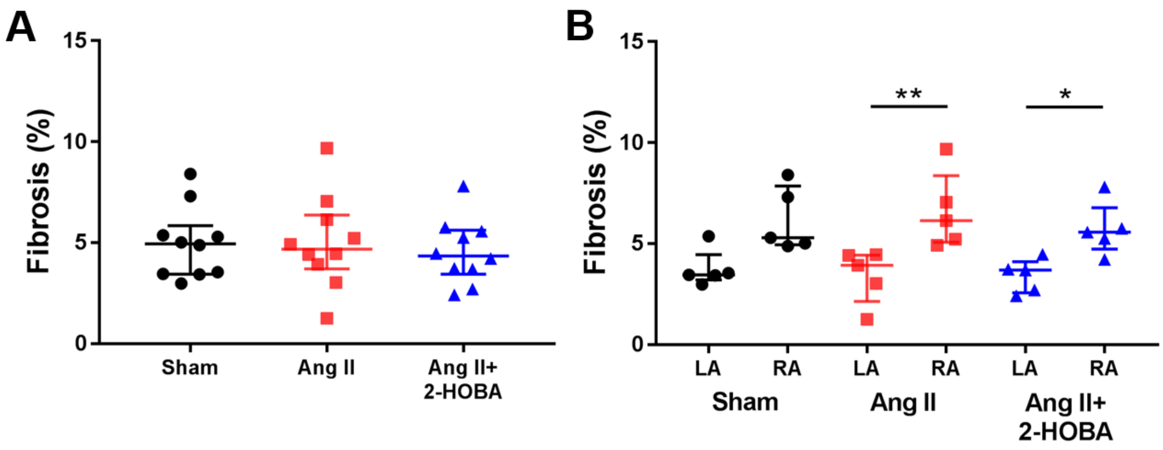

Supplement: Supplemental Figures 1 and 2 [file mmc1.pdf]
